# Completion pneumonectomy for lung cancer treatment: early and long term outcomes

**DOI:** 10.1186/1749-8090-7-107

**Published:** 2012-10-09

**Authors:** Peng Zhang, Chao Jiang, Wenxin He, Nan Song, Xiao Zhou, Gening Jiang

**Affiliations:** 1 Department of Thoracic Surgery, Shanghai Pulmonary Hospital, School of Medicine, Tongji University, Shanghai, Peoples Republic of China

**Keywords:** Lung cancer, Completion pneumonectomy, Follow-up

## Abstract

**Background:**

To analyze the experience of completion pneumonectomy for lung cancer at a single institution in China.

**Methods:**

From January 1988 to December 2007, 92 patients underwent completion pneumonectomy for the treatment of lung cancer. The indications were second primary lung cancer (n = 51), Local metastasis (n = 37) and Lung metastasis (n = 4). The median interval between the primary operation and CP was 24.4 months (1.5-145 m).

**Results:**

There was no intraoperative deaths. The CP procedure lasted 4.3 h (1.5-8 h). Blood loss in the CP performance was 1854.5 ml (200-9100 ml) 9 (9.78%) patients died in the postoperative period: pulmonary embolism (n = 2), disseminated intravascular coagulation (DIC) after the multisystem failure (n = 1), respiratory failure after contralateral pneumonia (n = 5), bronchopleural fistula (BPF) with acute respiratory distress syndrome (ARDS) (n = 1) 31(33.7%) patients had at least one major nonfatal complication. The 1, 3 and 5 year survival rates were 81%, 26% and 14% respectively.

**Conclusions:**

Completion pneumonectomy for lung cancer is a safe surgical procedure for the skilled surgeon though it has a relatively higher complications and the long-term survival is acceptable.

## Background

Completion Pneumonectomy is defined as the procedure to remove the reminder of the lung partially resected during a pervious operation [1]. When compared to standard pneumonectomy, it is a more challenging technical procedure associated with reported increased operative mortality and morbidity [2]. The indications for completion pneumonectomy include both benign and malignant diseases [3]. Indications for malignant disease have increased quickly with regard to the increasing frequency of lung cancer, the widespread use of limited resection for some early stage lung cancer and the increasing demands for pulmonary resection for pulmonary metastases. Indications for malignancies include local recurrence after prior lobectomy, second primary tumors, recurrent metastases from tumors in other systems or carcinoma occurring after previous lobectomy (or local resection) done for benign disease [1]. In this paper, we review our experience with this procedure for the treatment of lung cancer (CP for benign disease not being considered here) and to evaluate the postoperative outcomes and long-term results of it.

## Methods

### Definitions and inclusion criteria

The primary operation was defined as the first ipisilateral operation to move the lung tissue (eg lobectomy or bilobectomy or wedge resection). Hospital mortality included all intraoperative and postoperative deaths during hospitalization or within 30 days after the completion pneumonectomy but the patients were discharged earlier.

Recurrent lung cancer and second primary lung cancer were discriminated using the following criteria proposed by Martini and colleagues [4]: a second primary tumor was of different histology or if histology was the same, the disease-free interval between cancer was at least 2 years or the second cancer was in the different lobe but with no cancer in common lymphatic or extrapulmonary metastasis at the time of diagnosis or if its origin could be clearly traced to a carcinoma in situ.

### Patients

From January 1988 to December 2007, 92 patients underwent CP for the treatment of lung cancer at a single academic institution, which accounted for 4.5% of all pneumonectomy during the same period.

There were 83 male and 9 female patients. Mean age at the time of completion pneumonectomy was 57 years (32-71yo).There were 17 patients who underwent the primary operations at other hospitals.

The patients study variables included the following: age, sex, obesity (body mass index >27), smoking history, the indications for the primary operation and for CP, hypertention( systolic pressure >140 mmHg or diastolic pressure >90 mmHg or if the patient had already been treated for hypertention), pulmonary function, neoadjuvant treatment, interval between the primary operation and CP, TNM stage, the operative finding in CP, surgical procedure, operative mortality and long term survival. Written informed consent was obtained from the patient for publication of this report and any accompanying images.

The indications for the primary operation and for CP are showed as the Table 1.

**Table 1:** Indications for the primary operation and for CP

**Indicationfor the primary operation**	**No.**	**Indication for CP**	**No**
Non-small cell lung cancer	88	Second primary lung cancer	51
		Local metastasis	37
Tuberculosis	3	Lung metastasis	4
Bronchiectasis	1		

The 88 patients for lung cancer were sarcomatoid lung cancer (n = 1), small cell lung cancer (n = 2), squamous cell carcinoma (n = 46), adenocarcinoma (n = 29), adenosquamous carcinoma (n = 14). The 4 patients for benign disease: Tuberculosis (n = 3) and Bronchietasis (n = 1). The primary operation was lobectomy (n = 57), sleeve lobectomy (n = 15), lobectomy extended to the chest wall (n = 2) ,bilobectomy (n = 6) and segmentectomy (n = 12) and 15 patients were done the primary operation by VATS(video-assisted thoracic surgery). The non-small cell lung cancer had the TNM stage as the following: stage I (n = 53), stage II (n = 26), stage IIIa (n = 6), stage IIIb (n = 2) and stage IV (n = 1).After the first operation, 65 lung cancer patients had 4 cycles chemotherapy, 6 patients had the radiotherapy and 4 patients had both.

The median interval between the primary operation and CP was 24.4 months (1.5—145 m).

The indications for CP are showed in Table 1. The 88 patients with non-small cell lung cancer before were performed CP for: second primary lung cancer (n = 51) and local metastasis (n = 37). The 4 patients for the benign disease as the indications for the primary operation were performed CP for lung metastasis and they all had radical surgery before: breast cancer (n = 2), rectal cancer (n = 1) and endometrial cancer (n = 1).

Before CP, careful preoperative evaluation included assessment of the respiratory, cardiac and renal function. The patients was considered suitable for completion pneumonectomy if they had a predicted post-operative forced expiratory volume in 1 s (FEV1) of more than 1 l/s, estimated by spirometry exam and lung perfusion scan. Renal failure was considered as the contraindication.

All the patients were checked by chest CT scan, technetium bone scan and abdominal ultrasound. Mediastinoscopy was preformed when chest CT showed there was any mediastinal node with a mean diameter wider more than 1 cm in the recent 5 years. Bronchoscope examination was a standard procedure for all these patients. The above were used to evaluate if the patients had resectable diseases and they did not have regional or distant metastasis.

The postoperative TNM staging was: 4 Ia, 22 Ib, 3 IIa, 21 IIb, 30 IIIa, 7 IIIb, 5 IV (included 4 patients with lung metastasis and 1 patient with brain metastasis).

The primary end points of analysis were morbidity and mortality. The effect of risk factors on the end points were evaluated with both univariate and multivariate analysis. Risk factors represented by continuous variables were assessed by use of 2-sample t tests when the data were approximately normal and by rank sum tests when the data were found to be not sufficiently Gaussian. The effects of categorical variable risk factors were evaluated by means of X^2^test and the Fisher exact tests. Multiple logistic regression was used during multivariate analysis to simultaneously evaluate the effect of risk factors. P-value of the 0.05 or less was considered as significant. The Kaplan-Meier method, Life Tables and COX regression were used to evaluate the survival of the patients.

Follow-up was complete for all the patients in the group and data were obtained from the follow-up secretary in our department or from the government health agency.

## Results

CP was performed through a standard posterolateral approach with 5^th^ rib removed not considering the primary incision. There was no intraoperative deaths. The CP procedure lasted 4.3 h (1.5 h—8 h). Blood loss in the CP performance was 1854.5 ml (200—9100 ml), 65(70.6%) patients needed blood transfusion intra- or/ and postoperation (4.5 unite; range 1—23 unite), 9 (9.78%) patients died in the postoperative period: pulmonary embolism (n = 2), DIC after the multisystem failure (n = 1), respiratory failure after contralateral pneumonia (n = 5), BPF with acute respiratory distress syndrome (ARDS) (n = 1).

Factors associated with CP mortality in the univariate analysis included (as shown in Table 2): older age (>65 years ) (p = 0.012), obesity (p = 0.002) and preoperative radiotherapy (p < 0.001). But in the multivariate analysis, there were no any risk factor significantly associated with CP mortality.

**Table 2:** Factors associated with CP mortality

**Risk factors**	**P-value by univariate analysis**	**P-value by multivariate analysis**
Heavy smoker	ns	ns
Sex	ns	ns
Older age (>65 years old )	0.012	ns
Obesity	0.002	ns
CP at the right side	ns	ns
Hypertention	ns	ns
Diabets	ns	ns
Preoperative radiotherapy	<0.001	ns
Preoperative chemotherapy	ns	ns

31(33.7%) patients had at least one major nonfatal complication. These included respiratory insufficiency (6, 6.5%), heart failure (2, 2.2%), cardiac arrhythmia (12, 13.0%), hemorrhage necessitating reoperation (4, 4.3%), bronchopleural fistula(8,8.7%), empyema (3,3.3%), myocardial infarction(1,1.1%), sputum retention (6,6.5%), stress ulceration (2,2.2%).

BPF occurred in 8(8.7%) patients in this group,.1 patient died of it after ARDS and the other 7 were treated successfully by a Clagett procedure. Table 3 shows risk factors for the BPF.

**Table 3:** Risk factors for the BPF

**Risk factors**	**BPF**		**P value**
	**Yes**	**No**	
CP in the right side	7	54	ns
CP in the left side	1	30	
Hand sutured	2	59	0.016
With mechanical stapler	6	25	
Bronchial stump reinforcement	5	75	ns
Without reinforcement	3	9	
Diabetes	2	8	ns
Without Diabets	6	76	
Recurrence at the bronchial stump	1	24	ns
Not recurrence at the bronchial stump	7	60	
Preoperative radiation or chemotherapy	6	77	ns
Without preoperative radiation or chemotherpy	2	7	

**Figure 1:**
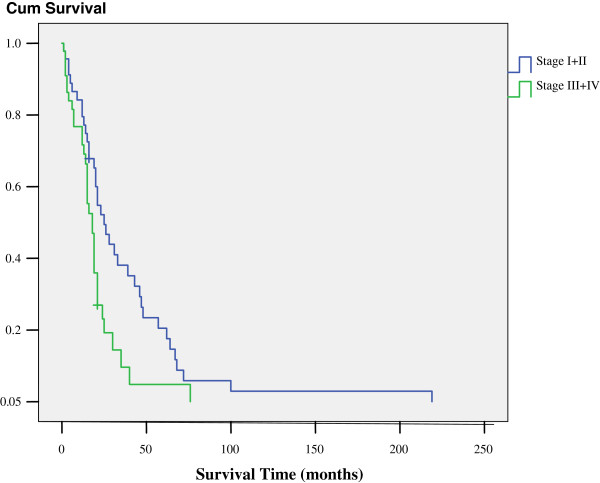
Survival time for stage I + II and stage III + IV lung cancer.

All the CP patients were completely follow-up. Mean follow-up time was 22.9 months (1-209 m). Actuarial 1-, 3- and 5 year survival rates from the time of CP were 81%, 26% and 14%. 5- year survival rates were similar in the subgroup with second primary lung cancer , recurrence lung cancer and lung metastases(p > 0.05). While there was significant difference between I + II stage and III + IV stage in the 5-year survival rate (20% vs 5%, P = 0.010, as shown in Figure 1). Risk factors associate with the 5-year survival rates are shown in the Table 4.

**Table 4:** Risk factors associated with the 5-year survival year rate of lung cancer

**Risk factor**	**Kaplan-Meier analysis**	**Cox Regression analysis**
sex	ns	ns
Right or left side	0.031	ns
Older age(>65 years old)	ns	ns
T	ns	ns
N	ns	ns
TNM stage	0.010	0.008
Second primary lung cancer or recurrence or lung metastases	ns	ns
Bronchial stump cancer left	ns	ns
The interval between the primary operation and CP	ns	ns

## Discussion

Completion pneumonectomy is really a challenging procedure for the thoracic surgeon. Sometimes it is really difficult for the thoracic surgeon to decide if the patient would benefit from the complex and dangerous procedure [5]. But if the patient has the lung cancer recurrence, or second lung cancer, or lung metastasis, to have the operation is the best choice for other treatments, such as chemotherapy and radiotherapy, could not bring a long survival time.

Indication of CP for malignant lung disease includes second primary lung cancer, local recurrence, recurrent metastases from tumors in other systems or carcinomas occurring after previous lobectomy for benign disease [1]. In our study, we had not collect the last group patients. Here, we are only interested in the result of CP for the second time cancer operation for malignant disease to see in such condition if CP was worthwhile.

CP is associated with high mortality and mobidity, which calls for careful planning before this procedure. Careful assessment should be made to reconsider if the patient has enough cardio-pulmonary reserve, the patient has a very good overall medical condition, and the patient does not have regional or distant metastasis. In the recent 5 years, in our department, repeat mediastinoscopy was sometimes used if the CT scan showed there were lymphonodes with the diameter more than 1 cm. It is also recommended to have a close study of the primary operation record: if the primary operation was done extrapleurally, if azygos vein had been cut down to explore the No 4 lymphonode, and how the bronchus stump dealt with, et al., which will help us to know what is the most difficulties in the following CP procedure.

CP for lung metastases is rarely used and analysed. There are only very limited data about CP for lung metastases. Three major reservations against the use of extended metastasectomy in the form of the CP [6]: The potentially higher perioperative morbidity and mortality associated with CP; A slightly improved long-term survival does not justify the increased perioperative risk; Extrapulmonary metastatic disease limited the long-term survival of patients. In our study, there were only 4 patients with CP for the lung metastases. It was too small number of the patients to draw a conclusion from it.

Intro-or postoperative bleeding is a very common affair for CP. In most series, the mean operative blood loss was more than 1 L. Postoperative hemorrhage requiring reoperation also often happened. In our study, the mean operative blood loss is 1854.3 l (200-9100 ml) and there were 4 postoperative hemorrhage requiring reoperation. It is similar to some other series. Such bleeding may come from taking down dense parietal adhesions or may originate from the direct injuries to the heart or pulmonary blood vessels. So it seems very important to get intrapericardial control of the pulmonary blood vessels in the operation. If the pericardial cavity is obliterated, one can divide the bronchus first, followed by ligation of the pulmonary artery and vein. Sometimes it is recommended to have mass closure of the hilar vessels and bronchus with transhilar horizontal mattress sutures, followed by oversewing of the vessels and bronchus. Watanable suggested CP could be performed by a median sternotomy. It was very easy to control the pulmonary vessels in such condition to lower the risk of hemorrhage. But on the other hand, it is really difficult to free dense adhesions and to resect chest wall if required. In our series, all the CP were performed through a standard posterolateral approach with 5^th^ rib removed not considering the primary incision.

For a majority of lung cancer patients who suffer from a recurrent or second primary lung cancer, CP offers the only chance for a cure. 5-year survival rate is one of the most important factors to evaluate the long-term results of CP. Compared with other articles, in our study the 5-year survival rate is relatively lower (as shown in Table 5). Maybe the following could explain it: In our study there were more later stage lung cancer (III + IV stage) patients who underwent CP procedure; All the patients at least had 2 operations for the cure of malignant disease, so their real overall condition were not very good.

**Table 5:** The results of CP for lung cancer in the literature

**Author**	**Years of publishing**	**Years of study**	**No.of lung cancer patients**	**Operative Mortality**	**5-year survival**
Neptune [7]	1966	----	8	12.5	---
Mathisen [8]	1984	1960-83	17	11.8	---
Nielsen [9]	1984	1974-82	4	0	---
McGovern [3]	1988	1958-85	64	9.4	26.4
Gregoire [10]	1993	1969-91	41	12.2	33
Terzi [11]	1994	1982-94	47	3.60	28.8
Massard [12]	1995	1978-82	21	0	23
AI-kattan [13]	1995	1980-93	21	0	23
Verhagen [14]	1996	1970-93	33	15.2	18.3
Regnard [15]	1999	1974-98	62	6.4	36
Fujimoto [1]	2001	1990-98	---	49	57
Miller [16]	2002	1985-98	58	17.6	--
Terzi [17]	2002	1982-2000	59	3.4	--
Guginno [18]	2004	1989-2002	46	11.9	44
Jungraithmayr [19]	2004	1986-2003	41	--	26
Chataigner [20]	2007	1996-2005	47	12.7	41
Current series	2008	1988-2007	94	9.8	14

## Conclusions

In summary, mortality and morbidity of CP for lung cancer are multifactorial and acceptable. Appropriate selection and meticulous perioperative care are paramount to minimize risk in those patients who require CP.

## Competing interests

The authors declare that they have no competing interests.

## Authors’ contributions

All authors read and approved the final manuscript.
